# Soyasaponins from Zolfino bean as aldose reductase differential inhibitors

**DOI:** 10.1080/14756366.2018.1553166

**Published:** 2018-12-27

**Authors:** Francesco Balestri, Marinella De Leo, Carlo Sorce, Mario Cappiello, Luca Quattrini, Roberta Moschini, Carlotta Pineschi, Alessandra Braca, Concettina La Motta, Federico Da Settimo, Antonella Del-Corso, Umberto Mura

**Affiliations:** a Department of Biology, University of Pisa, Pisa, Italy;; b Interdepartmental Research Center Nutrafood ‘‘Nutraceuticals and Food for Health’’, University of Pisa, Pisa, Italy;; c Department of Pharmacy, University of Pisa, Pisa, Italy;; d PhD School in Biochemistry and Molecular Biology, University of Siena, Siena, Italy

**Keywords:** Saponins, aldose reductase, differential inhibitors, Zolfino bean, *Phaseolus vulgaris*

## Abstract

Seven triterpenoid saponins were identified in methanolic extracts of seeds of the Zolfino bean landrace (*Phaseolus vulgaris* L.) by HPLC fractionation, revealing their ability to inhibit highly purified human recombinant aldose reductase (*h*AKR1B1). Six of these compounds were associated by MS analysis with the following saponins already reported in different *Phaseolus vulgaris* varieties: soyasaponin Ba (V), soyasaponin Bb, soyasaponin Bd (sandosaponin A), soyasaponin αg, 3-*O*-[R-l-rhamnopyranosyl(1 → 2)-*α*-d-glucopyranosyl(1 → 2)-*α*-d-glucuronopyranosyl]olean-12-en-22-oxo-3*α*,-24-diol, and soyasaponin βg. The inhibitory activity of the collected fractions containing the above compounds was tested for *h*AKR1B1-dependent reduction of both l-idose and 4-hydroxynonenal, revealing that some are able to differentially inhibit the enzyme. The present work also highlights the difficulties in the search for aldose reductase differential inhibitors (ARDIs) in mixtures due to the masking effect on ARDIs exerted by the presence of conventional aldose reductase inhibitors. The possibility of differential inhibition generated by a different inhibitory model of action of molecules on different substrates undergoing transformation is also discussed.

## Introduction

Saponins represent a wide family of compounds characterised by the presence of either a triterpenoid or a steroidal aglycone moiety and of one or more linked straight or branched sugar chains^1^. Saponins are widely distributed in higher plants, and both the aglycone and the saccharidic moieties of these molecules often define their natural origin and, to some extent, their functional properties and their industrial application^2^^,^^3^.

The amphiphilic nature of saponins provides these compounds with a significant biological action by enabling them to interact at the membrane level of the cell systems. In fact, they have been reported to act as anti-inflammatory, hypocholesterolemic, expectorant, vasoprotective, and immunomodulating agents^4^^,^^5^. Nonetheless, attention must be paid to the adverse cytotoxic action of saponins^6^^,^^7^. Indeed, this feature of saponins has been widely investigated as a potential tool to act against cell proliferation in cancer^8^.

As a result of their antioxidant and antidiabetic action, one of the emerging features of saponins is their ability to inhibit aldose reductase (AKR1B1). This enzyme (EC 1.1.1.21) catalyses the NADPH-dependent reduction of a wide range of hydrophilic as well as hydrophobic aldehydes. For several decades it has been investigated in order to find molecules able to inhibit its reducing activity on aldoses.

The rationale behind these investigations is based on the apparent link, in hyperglycaemic conditions, between sorbitol over-production and reducing power failure due to the AKR1B1 action and the development in diabetic subjects of pathological states such as retinopathy, nephropathy, peripheral neuropathy, cardiac dysfunctions, and cataracts^9^. Evidence of the effectiveness of yuchasaponins from the flower buds of *Camellia oleifera* as aldose reductase inhibitors (ARIs) on the rat lens enzyme has been reported^10^. More recently, *in vitro* inhibition of aldose reductase in a crude liver homogenate and *in vivo* inhibition of the enzyme in streptozotocin-induced diabetic rats, were reported for a furostanol saponin and its derivatives, extracted from *Balanites aegyptiaca*^11^^,^^12^. A triterpenoid oleanane saponin has also been reported to interfere with the polyol pathway through aldose reductase inhibition both *in vivo* in diabetic rats, and in an *in vitro* model of diabetic peripheral neuropathy^13^.

Although such reports in the literature highlight the ability of saponins to inhibit AKR1B1, to our knowledge no detailed kinetic study on these molecules has been carried out, and no evidence for their ability to act as aldose reductase differential inhibitors (ARDIs) has been reported.

Differential inhibition concerns the ability of a molecule to exert its inhibitory action depending on the nature of the substrate the enzyme is working on^14^. Thus, the ability to preferentially inhibit the reduction of sugar molecules with respect to hydrophobic aldehyde reduction makes ARDIs promising tools to counteract the development of secondary diabetic complications^15,16^.

In this work seven triterpenoid saponins were identified in methanolic extracts of seeds of the Zolfino bean landrace (*Phaseolus vulgaris* L.) revealing their ability to inhibit highly purified human recombinant *h*AKR1B1 and, for some of them, to differentially inhibit the enzyme depending on the substrate (l-idose or 4-hydroxynonenal, HNE) undergoing reduction.

## Materials and methods

### Materials

Bovine serum albumin (BSA), d,l-dithiothreitol (DTT), d,l-glyceraldehyde (GAL), EDTA and DSC18 hydrophobic interaction cartridges Supelco Discovery were purchased from Sigma-Aldrich (Saint Louis, MO, USA). NADPH, l-idose, soyasaponin Ba (SSBa) and soyasaponin Bb (SSBb) were supplied by Carbosynth (Compton, England); YM10 ultrafiltration membranes were obtained from Merck-Millipore (Darmstad, Germany); soyasaponin Bd (SSBd) was obtained from ALB Technology Limited (Mongkok Kowloon, Hong Kong); PTFE filtration membranes 0.45 µm pore size were from Phenomenex Italy (Bologna, Italy); HPLC grade methanol, formic acid, and acetic acid were purchased from VWR (Milano, Italy). HPLC grade water (18 MΩ) was prepared by a Mill-Ω purification system (Millipore Corp., Bedford, MA). All other chemicals were of reagent grade.

Dry seeds of yellow Zolfino landrace were obtained from the farm Agostinelli Mario in Leccio-Reggello (Florence, Italy; 43° 42' N, 11° 27' E) and authenticity was confirmed by comparing their features with those registered in the “Regione Toscana” germplasm data bank (access VE_027): http://germoplasma.arsia.toscana.it/.

### Preparation of crude extracts from bean seeds

Dried bean seeds, mechanically disrupted by Ultra-Turrax, were subjected to extraction by the addition (5 ml/g) of 80% methanol aqueous solution containing 0.6% (v/v) of acetic acid. The suspension was stirred for approximately 5 h at 4 °C temperature in darkness, centrifuged for 10 min at 4 °C at 7000 *g*, and the pellet extracted again at 4 °C overnight as above. All the solvents used in the subsequent manipulation of the extract (water or methanol solutions in water) contained 0.6% (v/v) final concentration of acetic acid. The supernatants of the two centrifugation steps were pooled, dried at room temperature by rotary evaporator, resuspended (2 ml/g equivalents of the initial dry seeds) in 10% methanol aqueous solution and filtered through 0.45 µm PTFE membrane filters.

### Preparation and fractionation of “Zolfino bean-enriched extract”

On the basis of previous results of the fractionation of Zolfino extract on a reverse phase HPLC column through a methanol/aqueous acetic acid gradient^16^, the extract was enriched in those components with the highest hydrophobicity.

Thus, the extract was applied on 5 ml DSC18 hydrophobic interaction cartridges (1 ml sorbent bed) previously conditioned by sequential addition of 5 ml of the following solutions (each containing 0.6% acetic acid v/v): 100% methanol, water and 10% methanol. A volume of 2.5 ml of bean extract was applied to the cartridge, and a stepwise elution was performed by the sequential addition of 5 ml of the following solutions: water, 10%, 30%, 50%, and 100% methanol. The fraction eluting with 100% methanol was concentrated by a rotary evaporator, diluted with water (containing 0.6% acetic acid) to obtain 40% methanol in a final volume of 500 μl, and filtered through a 0.45 µm PTFE membrane. The sample obtained is referred to hereafter as the “enriched extract”.

The “enriched extract” was fractionated by a SpectraSystem HPLC instrument (Thermo, Rodano, Italy), equipped with a Kinetex C18 column, 250 × 4.6 mm ID, 5 µm particle size (Phenomenex, Bologna, Italy). A mixture of methanol with 0.6% acetic acid (solvent A) and a 0.6% v/v aqueous solution of acetic acid (solvent B) were used as the eluent. The gradient profile was as follows: 0–6 min, 40% A, isocratic mode; 6–26 min, 40–100% A; 26–36 min, 100% A, isocratic mode. Elution was performed at a flow rate of 1 ml/min; the absorbance at 254 nm was monitored online. The column was loaded with samples of about 1 g equivalent of the initial dry seed weight per run. The eluate was collected by splitting it into 18 fractions, 2 ml each.

Individual fractions (F1–18) were dried and resuspended in 0.1 ml methanol and assayed (15 µl) for *h*AKR1B1 inhibition ability.

Aliquots of the HPLC fraction eluted with a retention time (*t*_R_) from 24 to 26 min (F13) were dried, resuspended in 55% methanol with 0.6% acetic acid, and filtered through 0.45 µm PTFE membrane. They were further purified by HPLC in the same conditions described above, except for the elution programme, which was as follows: 0–4 min, 55% A, isocratic mode; 4–34 min, 55–100% A; 34–44 min, 100% A, isocratic mode. The solvent eluting from the column between 22 and 28 min was split into fractions, which were named F13a, F13b and so on, and assayed for *h*AKR1B1 inhibition ability. In order to optimise the purification of the putative compounds with differential inhibition activity, the number of fractions collected and the pattern of separation was fine-tuned on the basis of the results of the bioassay. This led to the isolation of five fractions (F13a–F13e), which were collected according to their chromatographic profile thereby obtaining sub-fractions F13a–e.

### HPLC-PDA/UV-ESI-MS/MS analyses

HPLC-photodiode array (PDA)/UV-electrospray ionisation (ESI)-tandem mass spectrometry (MS/MS) analyses were performed using a Surveyor LC pump, a Surveyor autosampler, coupled with a Surveyor PDA detector, and an LCQ Advantage ion trap mass spectrometer (Thermo/Finnigan, San Jose, CA, USA) equipped with Xcalibur 3.1 software. Analyses were performed on a 4.6 × 150 mm, 4 µm, Synergi Fusion-RP column (Phenomenex Italy, Bologna, Italy), using a mixture of methanol (solvent A) and a 0.05% v/v aqueous solution of formic acid (solvent B) as the eluent.

The gradient profile was as follows: 0–6 min, 40% A, isocratic mode; 6–28 min, 40–100% A; 28–36 min 100% A, isocratic mode. Elution was performed at a flow rate of 0.8 ml/min with a splitting system of 2:8 to the MS detector (160 μl/min) and the PDA detector (640 μl/min), respectively. MS/MS analyses were performed with an ESI interface in positive ion mode with a scan range of *m/z* 150–2000, using N_2_ as the sheath and auxiliary gas.

The parameters used for MS operating conditions were optimised as follows: capillary temperature, 270 °C; sheath gas flow rate, 60.00 arbitrary units; auxiliary gas flow rate, 3.00 arbitrary units; capillary voltage, 32.00 V; tube lens offset, 10.00 V; spray voltage, 4.50 kV. PDA data were recorded with a 200–600 nm range. Analysed fractions 13a–e were first dried using a Speedvac concentrator, then dissolved in methanol at a final concentration of 2.0 mg/ml and centrifuged; a volume of 20 μl of supernatants was injected into the LC-MS system.

### Assay of aldose reductase

The AKR1B1 activity was determined at 37 °C as previously described^17^, monitoring the decrease in absorbance at 340 nm linked to NADPH oxidation (ε_340_=6.22 mM^−1 ^cm^−1^) through a Biochrom Libra S60 spectrophotometer. In a 0.25 M sodium phosphate buffer pH 6.8, the standard assay mixture contained 0.18 mM NADPH, 0.4 M ammonium sulphate, 0.5 mM EDTA and 4.7 mM GAL. One unit of enzyme activity is the amount that catalyses the conversion of 1 µmol of substrate/min in the above assay conditions. These assay conditions were also adopted to assess the effectiveness of inhibitors when l-idose or HNE were used, at the indicated concentrations, as substrates instead of GAL. Differential inhibition (DI) refers to the difference between the percentage inhibition observed using l-idose and HNE as substrates in the assay conditions indicated.

### Purification of human recombinant AKR1B1

The human recombinant AKR1B1 (*h*AKR1B1) was expressed and purified to electrophoretic homogeneity as previously described^18^. The purified enzyme (specific activity 5.3 U/mg) was stored at −80 °C in a 10 mM sodium phosphate buffer pH 7.0 containing 2 mM DTT and 30% (w/v) glycerol. Before use, the enzyme was extensively dialysed against a 10 mM sodium phosphate buffer pH 7.0.

### Other methods

The protein concentration was determined by the Coomassie blue staining method^19^, using BSA as a standard protein. Statistical analysis was performed using the two-way ANOVA test carried out with Graphpad 6.0 software. IC_50_ determination was performed using standard statistical software (GraphPad Instat version 6.0, San Diego, CA). Results are reported as the mean of the values and the 95% confidence interval from at least three independent measurements.

## Results and discussion

**Figure 1.**
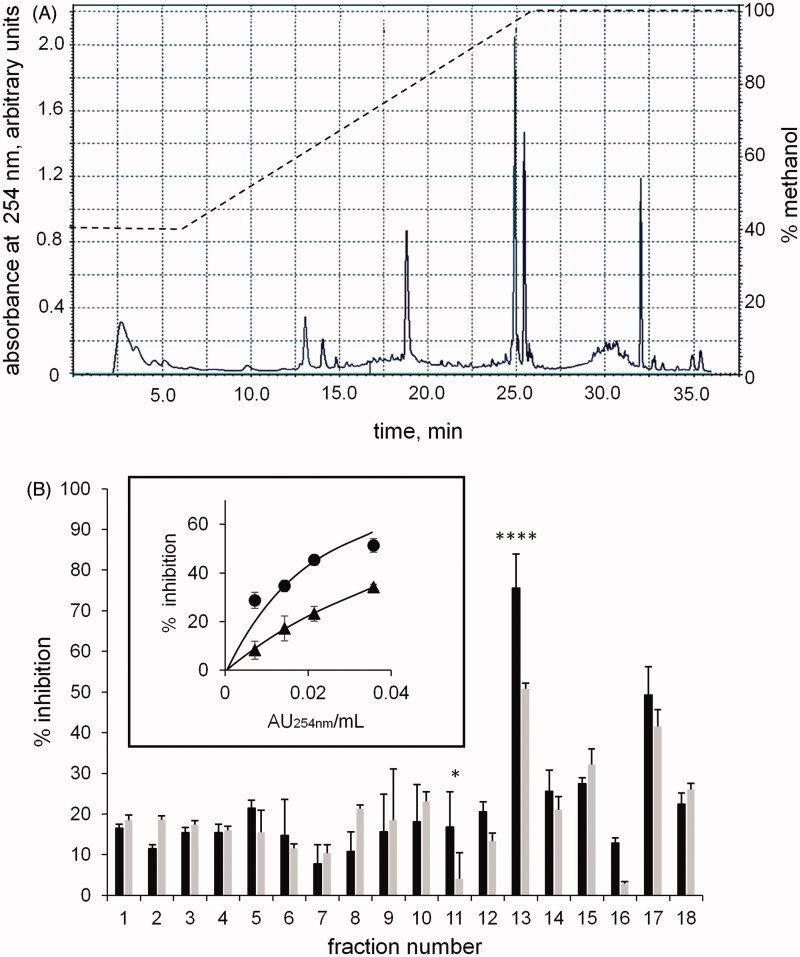
Chromatographic fractionation of the hydrophobic components-enriched Zolfino bean extract. *Panel* A: the separation profile monitored at 254 nm of the enriched extract applied (0.5 ml) on the C18 column and eluted by a methanol-aqueous acetic acid gradient as indicated in the figure by a dotted line (see Materials and Methods for details). *Panel* B: the percentage of inhibition exerted by the collected individual fractions (2 ml) on the l-idose (black bars) and HNE (gray bars) reduction. Eight mU of *h*AKR1B1 were used in the assay with 0.8 mM and 0.04 mM of l-idose and HNE, respectively, as substrates (see Materials and Methods for details). *Pane*l B (inset): the inhibitory action in the above assay conditions (% inhibition) exerted by different amounts of HPLC fraction F13 (AU_254nm_/mL assay) on the *h*AKR1B1 catalysed reduction of l-idose (circles) and HNE (triangles) used as substrates. Error bars (when not visible are within the symbol size) represent the standard deviations of the mean from at least three independent measurements. The statistical significance of differential inhibition % on l-idose reduction with respect to HNE reduction is reported as: ****p < 0.0001 and *p < 0.05.

We recently reported that components of a methanolic extract of Zolfino bean, eluting as the most hydrophobic fractions from an HPLC reverse phase column, act as a promising source of ARDIs^16^. Thus in the present work, a methanolic extract enriched in these components was fractionated by reverse phase HPLC (see Methods), and the relative elution profile and the differential inhibitory activity of the eluted fractions (F1–18) are reported in Figure 1(A,B), respectively.

Components eluting with *t*_R_ from 24 to 26 min (Figure 1, F13), which also showed the highest effectiveness as ARIs and the most appreciable differential inhibitory action, were further investigated. The inset of Figure 1 reports the inhibitory action on both l-idose and HNE reduction versus the amount of F13 (expressed as absorbance units at 254 nm/ml assay). The differential inhibitory activity of the sample with respect to the reduction of the two substrates with an IC_50_ for HNE (0.069 AU_254 nm_/ml assay, with 0.059–0.080, 95% confidence limits) was clear, and was approximately two fold higher (*p < 0.05) than that measured for l-idose (IC_50_=0.029 AU_254 nm_/ml assay, with 0.023–0.036, 95% confidence limits).

**Figure 2.**
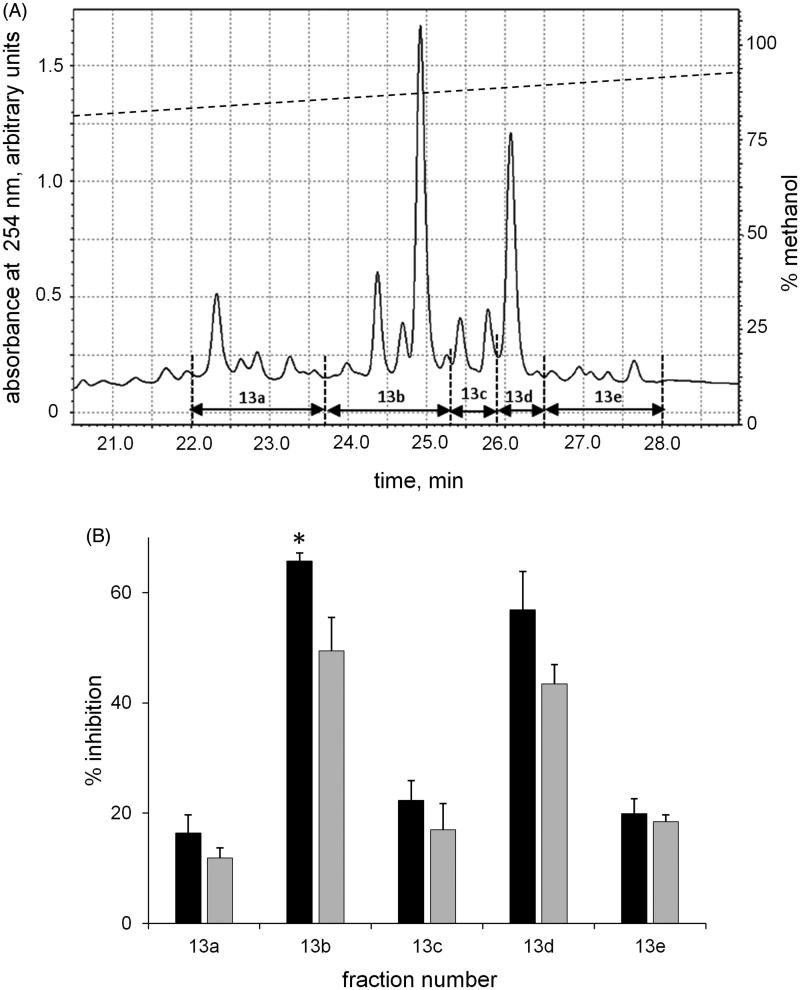
Chromatographic fractionation of F13. The column eluate with *t*_R_ from 24–26 min (Figure 1, F13) from three different runs was pooled, dried, resuspended in 0.5 ml of 55% methanol in 0.6% v/v aqueous solution of acetic acid and subjected to chromatographic separation in the conditions described in the Materials and Methods. *Panel* A: reports the elution profile at 254 nm and the manually collected eluting fractions (namely 13 a–13 e). *Panel* B: the inhibitory ability of the collected fractions on the *h*AKR1B1 catalysed reduction of l-idose (black bars) and HNE (gray bars). Error bars (when not visible are within the symbol size) represent the standard deviations of the mean from at least three independent measurements. The statistical significance of differential inhibition % on l-idose reduction with respect to HNE reduction is reported as: *p < 0.05.

A further HPLC fractionation of F13 (see Methods) separated the suitable sub-fractions for the evaluation of the inhibitory capacity and for component identification. Thus, fractions with *t*_R_ from 22 to 28 min, namely 13a–13e (Figure 2, *Panel* A), were manually collected, dried and analysed for differential inhibitory ability (Figure 2, *Panel* B). As reported in Figure 2, the elution components showed a different ability in differentially inhibiting *h*AKR1B1. Fractions 13 b and 13d appeared to be the most powerful inhibitors, with a comparable effectiveness in inhibiting l-idose reduction. However, when considering their differential inhibitory ability (DI) between l-idose and HNE, despite comparable values (16.78 ± 7.5% and 13.41 ± 6.4% for 13 b and 13d, respectively), statistical significance (*p values <0.05) was observed only for data referring to 13 b. The remaining three fractions (namely 13a, 13c and 13e) showed a lower inhibitory capacity and no significant evidence of differential inhibition.

In order to reveal the chemical structures of their bioactive components, sub-fractions 13a–e were analysed by means of HPLC-PDA/UV-ESI-MS/MS techniques.

**Figure 3.**
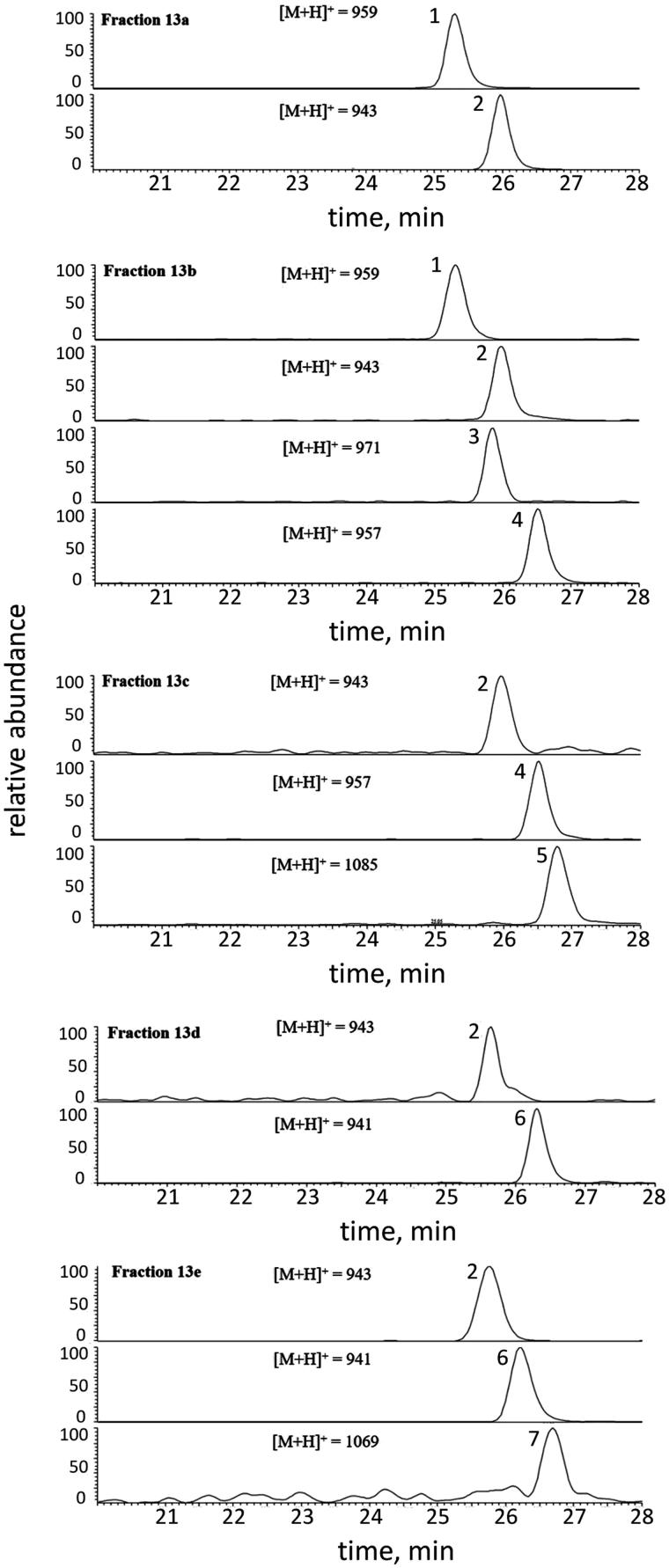
Extracted ion chromatograms of saponins **1**–**7** detected in fractions 13 a–e by LC-ESI-MS/MS analysis, registered in positive ion mode. **1 **=** **Soyasaponin Ba; **2 **=** **Soyasaponin Bb; **3 **=** **Unidentified; **4 **=** **Soyasaponin Bd; **5 **=** **Soyasaponin αg; **6 **=** **3-*O*-[R-l-rhamnopyranosyl(1 → 2)-*α*-d-glucopyranosyl(1 → 2)-*α*-d-glucuronopyranosyl]olean-12-en-22-oxo-3*α*,-24-diol; **7 **=** **Soyasaponin βg. Peak data are listed in Table 1. The structures of identified compounds are shown in Figure 4.

**Figure 4.**
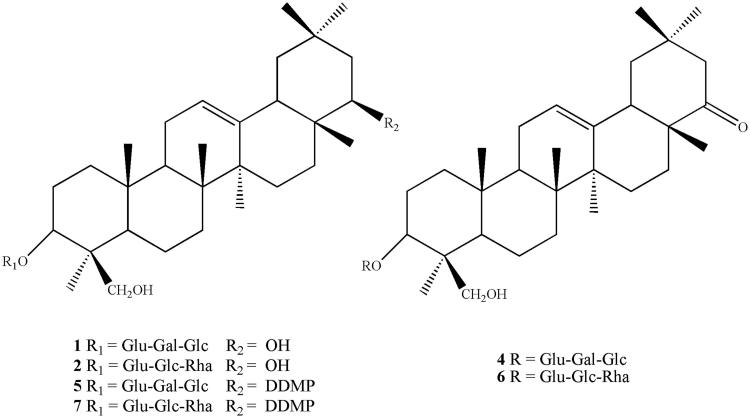
Structures of saponins **1**, **2**, and **4**–**7** detected in sub-fractions 13 a–e. The compound distribution into the fractions is shown in Figure 3.

The results showed the presence of triterpenoid saponins (**1**–**7**) in all the analysed fractions, whose peak distribution (identified with the same corresponding numbers) is illustrated in Figure 3. All compounds, except for compound **3**, were identified by comparing their spectral data (UV and ESI-MS/MS) with those reported in the literature^20–22^. In addition, compounds **1** and **2** were compared with authentic pure standards. Thus, the structures were assigned as follows: soyasaponin Ba (V), SSBa (**1**), soyasaponin Bb (I), SSBb (**2**), soyasaponin Bd (sandosaponin A), SSBd (**4**), soyasaponin αg, SS αg (**5**), 3-*O*-[R-l-rhamnopyranosyl(1 → 2)-*α*-d-glucopyranosyl(1 → 2)-*α*-d-glucuronopyranosyl]olean-12-en-22-oxo-3*α*,-24-diol (**6**), and soyasaponin βg, SS βg (**7**) (Figure 4). All saponins identified had previously been isolated from *Phaseolus vulgaris*^20^^,^^23^^,^^24^, whereas compound **3** remained unidentified.

The molecular weights of all the constituents were deduced from the presence in the full mass spectra, acquired in the positive ion mode, of both protonated [M + H]^+^ and sodiated [M + Na]^+^ ion molecular peaks. The analysis of the fragmentation patterns led to the identification of all the molecules as soyasaponins B and E, with two different aglycones (soyasapogenol B and E) and two different trisaccharide sugar chains attached to the C-3 hydroxyl position of the aglycone moieties. Furthermore, saponins **5** and **7** showed the sugar 2,3-dihydro-2,5-dihydroxy-6-methyl-4*H*-pyran-4-one (DDMP) attached to the C-22 hydroxyl group.

The ESI-MS/MS of sodium adduct ions (Table 1) of compound **1** ([M + Na]^+^ at *m/z* 981), compound **4** ([M + Na]^+^ at *m/z* 979), and compound **5** ([M + Na]^+^at *m/z* 1107), showed very similar fragmentation pathways, with diagnostic peaks at *m/z* 819, 657, and 481 (compound **1**), *m/z* 817, 655, and 479 (compound **4**), and *m/z* 945, 783, and 607 (compound **5**) due to the sequential losses of one glucose ([M − 162 + Na]^+^]), one galactose ([M − 162 − 162 + Na]^+^]), and one glucuronic acid ([M − 162 − 162 − 176 + Na]^+^]) residues, respectively, thus confirming the presence of a trisaccharide chain linked to the aglycones. According to common fragmentation patterns observed for saponins^25^, the MS/MS spectra also showed signals corresponding to a sodium-cationised sugar chain, such as the ion peaks at *m/z* 541 comprised of glucose, galactose, and glucuronic acid ([162 + 162 + 194 + Na]^+^), detected for the three compounds. In addition, other fragments corresponding to the trisaccharide chain sodium adduct ions were detected, due to the elimination of one molecule of water [162 + 162 + 194 – 18 + Na]^+^, a carboxylic residue [162 + 162 + 194 – 18 – 44 + Na]^+^, and the glucuronic acid unit [162 + 180 + Na]^+^. Thus, compounds **1** and **4** were identified as SSBa and SSBd, which differed in the aglycone moieties represented by the soyasapogenol B and soyasapogenol E, respectively. Compared to **1**, compound **5** showed in addition a residue of 126 amu corresponding to a DDMP unit, and thus was characterised as soyasaponin αg.

**Table 1.** ESI-MS/MS and chromatographic data (retention time, *t*_R_) of triterpenoid saponins **1**–**7** detected in the fractions 13 a–e from *Phaseolus vulgaris*.

Peak^a^	Saponins	*t*_R_ (min)	[M + H]^+^ (*m/z*)	[M + Na]^+^ (*m/z*)	MS/MS ions (*m/z*)^b^/ Assignments
**1**	Soyasaponin Ba (V)	25.3	959	981	819/[M − Glc + Na]^+^ 657/[M − Glc − Gal + Na]^+^ 481/[M − Glc − Gal − Glu + Na]^+^ 541/[Glc + Gal + Glu + Na]^+^ 523/[Glc + Gal + Glu–H_2_O + Na]^+^ 505/[Glc + Gal + Glu–2H_2_O + Na]^+^ 461/[Glc + Gal + Glu–2H_2_O-CO_2_+Na]^+^ 443/[Glc + Gal + Glu–3H_2_O-CO_2_+Na]^+^ 347/[Glc + Gal–H_2_O + Na]^+^
**2**	Soyasaponin Bb (I)	26.0	943	965	819/[M − Rha + Na]^+^ 657/[M − Rha − Glc + Na]^+^ 481/[M − Rha − Glc − Glu + Na]^+^ 525/[Rha + Glc + Glu + Na]^+^ 507/[Rha + Glc + Glu–H_2_O + Na]^+^ 489/[Rha + Glc + Glu–2H_2_O + Na]^+^ 445/[Rha + Glc + Glu–2H_2_O-CO_2_+Na]^+^ 427/[Rha + Glc + Glu–3H_2_O-CO_2_+Na]^+^ 331/[Rha + Glc–H_2_O + Na]^+^
**3**	Unidentified	25.8	971	993	831/[M − Hex + Na]^+^ 669/[M − 2Hex + Na]^+^ 555/[Saccharide chain + Na]^+^
**4**	Soyasaponin Bd (sandosaponin A)	26.5	957	979	817/[M − Glc + Na]^+^ 655/[M − Glc − Gal + Na]^+^ 479/[M − Glc − Gal − Glu + Na]^+^ 541/[Glc + Gal + Glu + Na]^+^ 523/[Glc + Gal + Glu–H_2_O + Na]^+^ 505/[Glc + Gal + Glu–2H_2_O + Na]^+^ 461/[Glc + Gal + Glu–2H_2_O-CO_2_+Na]^+^ 443/[Glc + Gal + Glu–3H_2_O-CO_2_+Na]^+^ 347/[Glc + Gal–H_2_O + Na]^+^
**5**	Soyasaponin αg	26.8	1085	1107	945/[M − Glc + Na]^+^ 783/[M − Glc − Gal + Na]^+^ 607/[M − Glc − Gal − Glu + Na]^+^ 523/[Glc + Gal + Glu–H_2_O + Na]^+^ 505/[Glc + Gal + Glu–2H_2_O + Na]^+^ 461/[Glc + Gal + Glu–2H_2_O-CO_2_+Na]^+^ 443/[Glc + Gal + Glu–3H_2_O-CO_2_+Na]^+^ 347/[Glc + Gal–H_2_O + Na]^+^
**6**	3-*O*-[R-l-rhamnopyranosyl(1→2)-*α*-d-glucopyranosyl(1→2)-*α*-d-glucuronopyranosyl]olean-12-en-22-oxo-3*α*,-24-diol.	26.3	941	963	817/[M − Rha + Na]^+^ 655/[M − Rha − Glc + Na]^+^ 479/[M − Rha − Glc − Glu + Na]^+^ 525/[Rha + Glc + Glu + Na]^+^ 507/[Rha + Glc + Glu–H_2_O + Na]^+^ 489/[Rha + Glc + Glu–2H_2_O + Na]^+^ 445/[Rha + Glc + Glu–2H_2_O-CO_2_+Na]^+^ 427/[Rha + Glc + Glu–3H_2_O-CO_2_+Na]^+^ 331/[Rha + Glc–H_2_O + Na]^+^
**7**	Soyasaponin βg	26.7	1069	1091	945/[M − Rha + Na]^+^ 783/[M − Rha − Glc + Na]^+^ 607/[M − Rha − Glc − Glu + Na]^+^ 525/[Rha + Glc + Glu + Na]^+^ 507/[Rha + Glc + Glu–H_2_O + Na]^+^ 489/[Rha + Glc + Glu–2H_2_O + Na]^+^ 445/[Rha + Glc + Glu–2H_2_O-CO_2_+Na]^+^ 427/[Rha + Glc + Glu–3H_2_O-CO_2_+Na]^+^ 331/ [Rha + Glc–H_2_O + Na]^+^

^a^Compound numbers correspond with peak numbers in Figure 3. ^b^MS/MS data are obtained from the fragmentation of the [M + Na]^+^ precursor ions. Gal: galactose; Glc: glucose; Glu: glucuronic acid; Hex: hexose; Rha: rhamnose.

The full mass spectra of compounds **2**, **6**, and **7** showed sodium adduct molecular ion peaks [M + Na]^+^ at *m/z* 965, 963, and 1091, respectively.

Ion peaks corresponding to the subsequent losses of rhamnose [M–146 + Na]^+^, glucose [M–146 − 162 + Na]^+^, and glucuronic acid [M–146 − 162 − 176 + Na]^+^ units can be observed in the fragmentation patterns of all three precursor ions, indicating the presence of the same trisaccharide chain in these three saponins. This evidence was confirmed by the detection of fragments at *m/z* 507 ([146 + 162 + 194 + Na]^+^), 489 ([146 + 162 + 194 – 18 + Na]^+^), 445 ([146 + 162 + 194 – 18 – 44 + Na]^+^), 427 ([146 + 162 + 194 – 18 – 18 − 44 + Na]^+^), and 331 ([146 + 180 + Na]^+^) related to the glycosidic portion.

**Figure 5.**
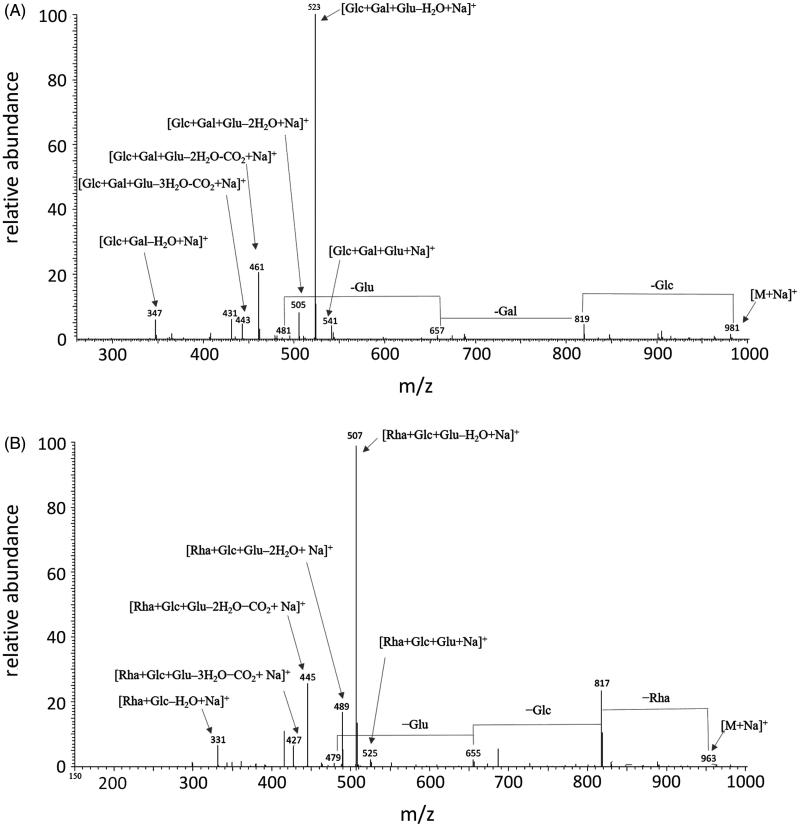
ESI-MS/MS spectra of precursor ions [M + Na]^+^ of saponins **1** (*Panel* A) and **6** (*Panel* B), differing in the aglycone and in the saccharide portion. Gal: galactose; Glc: glucose; Glu: glucuronic acid; Rha: rhamnose.

The aglycones were established as soyasapogenol B for compound **2** and soyasapogenol E for compound **6**, as deduced from the presence of product ion peaks at *m/z* 479 and 481, respectively. Thus, compounds **2** and **6** were identified as SSSBb and 3-*O*-[R-l-rhamnopyranosyl(1→2)-*α*-d-glucopyranosyl(1→2)-*α*-d-glucuronopyranosyl]olean-12-en-22-oxo-3*α*,-24-diol, respectively. In addition, compound **7** was characterised by the presence of a DDMP residue and the same aglycone of **2**, leading its structure being identified as soyasaponin βg. As an example, the MS/MS spectra of compounds **1** and **6** are shown in Figure 5. Finally, compound **3**, which was detected in F13b coeluting with compound **2**, showed a molecular weight of 970 amu, as deduced from both the sodium adduct [M + Na]^+^ and protonated [M + H]^+^ molecular ion peaks at *m/z* 993 and 971, respectively.

The analysis of the fragmentation pathway of *m/z* 993 precursor ion suggested the presence of two hexose residues (peaks at *m/z* at 831 [M − 162 + Na]^+^ and 669 [M − 162 − 162 + Na]^+^) in the saccharide chain. However, the detection of the peak at *m/z* 609 ([M − 162 − 162 − 60 + Na]^+^) suggested the presence of an acetyl group. On the other hand, the base peak was represented by a product ion at *m/z* 555, related to the oligosaccharide chain, thus differing from both glycosidic portions linked to the other identified substances. Although the MS/MS analyses clearly indicated that compound **3** was a triterpenoid saponin related to the other analysed molecules, the complete assignment of its structure was not possible on the basis of the MS data. Thus, compound **3** remained unidentified.

As highlighted by the LC-MS analyses (Figure 3), the investigated fractions (F13a–F13e) were a mixture of two or more compounds. Referring to the inhibition data (Figure 2), the most interesting fraction of the Zolfino extract was F13b in which the MS analysis revealed, besides SSBa and SSBb, the presence of SSBd (also present in F13c) and, only for this fraction, of a yet unidentified saponin (peak **3** in Figure 3) with a molecular mass of 970 amu. This made it difficult to unequivocally associate the specific saponin species with their differential inhibitory features. Nevertheless an association was attempted. Compound **1**, whose presence was revealed in both F13a and F13b, and compound **2**, which was detected from fraction F13a to F13e, did not seem to exert any differential inhibitory action. This was evident from looking at the inhibition data of F13a in which these two compounds predominate. Similarly, the fact that fractions 13c, 13d and 13e, did not exhibit a significant differential inhibition, led the initial conclusion that also compounds **5–7** appeared to be devoid of a differential inhibitory capacity. This left the unidentified compound **3** and possibly compound **4** (SSBd) as the only potential ARDIs conferring differential inhibition to F13b.

Such a conclusion, however, is debateable. In fact, the simultaneous presence in a mixture of more than one component capable of inhibiting the enzyme, may mask the possible presence of an ARDI. In this regard, the couple of compounds **1** and **2** is indicative of the difficulty in searching for differential inhibitors in multi-component mixtures. In this case, the availability of authentic standards of compounds **1** and **2** identified in the Zolfino bean (SSBa and SSBb, respectively) facilitated a detailed kinetic investigation in order to gain insights into the inhibitory features of these two unresolved components present in F13a.

**Figure 6.**
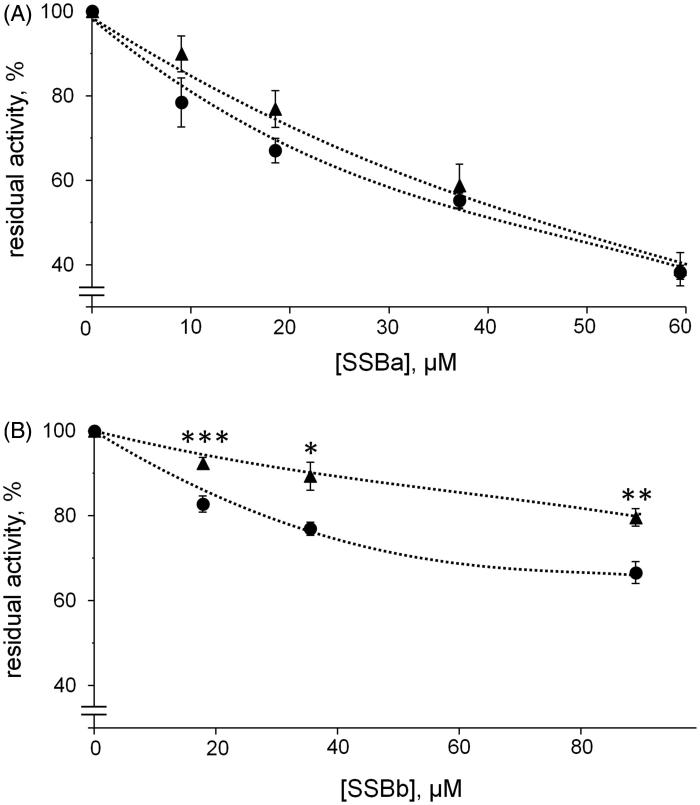
Inhibition curves of *h*AKR1B1 by soyasaponins. Authentic standards of SSBa (*Panel* A) and SSBb (*Panel* B) were used to evaluate the dose-dependent inhibitory effect on 8 mU of the enzyme acting either on 0.8 mM of l-idose (circles) or 0.04 mM of HNE (triangles). Error bars (when not visible are within the symbol size) represent the standard deviations of the mean from three to five independent measurements. Asterisk(s) indicate significant differences between l-idose and HNE (*p ≤ 0.05, **p ≤ 0.01, ***p ≤ 0.001).

To compare inhibition curves of different substrates, the substrates concentration must be kept at values equal or equally proportional to their respective *KM* values. This to allow the enzyme to act on each substrate in comparable conditions. In the present study the concentrations of the two used substrates were chosen being close to their *KM* values, i.e. 0.04 mM for HNE^17^ and 0.8 mM for l-idose^18^. The results reported in Figure 6 indicated that SSBa behaved like an ARI since it was able to inhibit l-idose and HNE reaction with essentially the same effectiveness, with an IC_50_ of 40 µM (34–47 µM, 95% confidence limits) and 47 µM (37–59 µM, 95% confidence limits), respectively. Conversely, SSBb, although less efficient than SSBa as an inhibitor, showed a differential inhibitory action between l-idose and HNE, since the IC_50_ for HNE reduction (360 µM; 290–440 µM 95% confidence limits) was two fold higher than that for l-idose reduction (IC_50_ 170 µM; 130–220 µM, 95% confidential limit).

**Figure 7.**
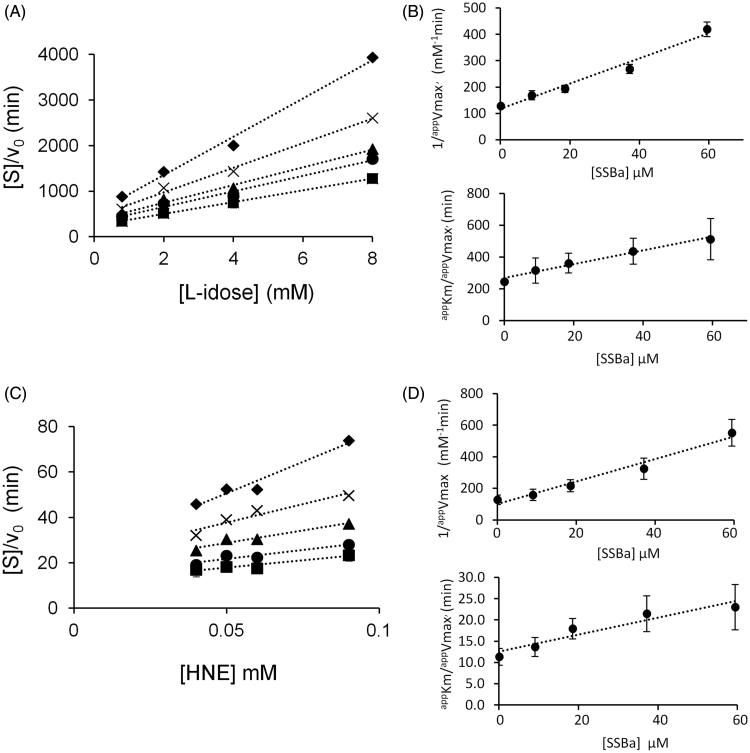
Kinetic characterisation of SSBa as *h*AKR1B1 inhibitor. *Panel* A and *Panel* C are the Hanes–Woolf plots obtained when the activity of the purified enzyme (8 mU) was measured at the indicated concentrations of the substrate, in the absence (■) or in the presence of the following inhibitor concentrations: (●) 9 µM, (▴) 18.5 µM, (×) 37.1 µM, (♦) 59.4 µM. *Panel* B and *Panel* D refer to the secondary plots of the slopes (1/*^app^V_max_*) and the ordinate intercept (*^app^K_m_*/*^app^V_max_*) of the relative Hanes–Woolf plot, as a function of the inhibitor concentration. *Panel* A and *Panel* B refer to l-idose; *Panel* C and *Panel* D refer to HNE. Error bars (when not visible are within the symbols size) represent the standard deviations of the mean from at least three independent measurements.

**Figure 8.**
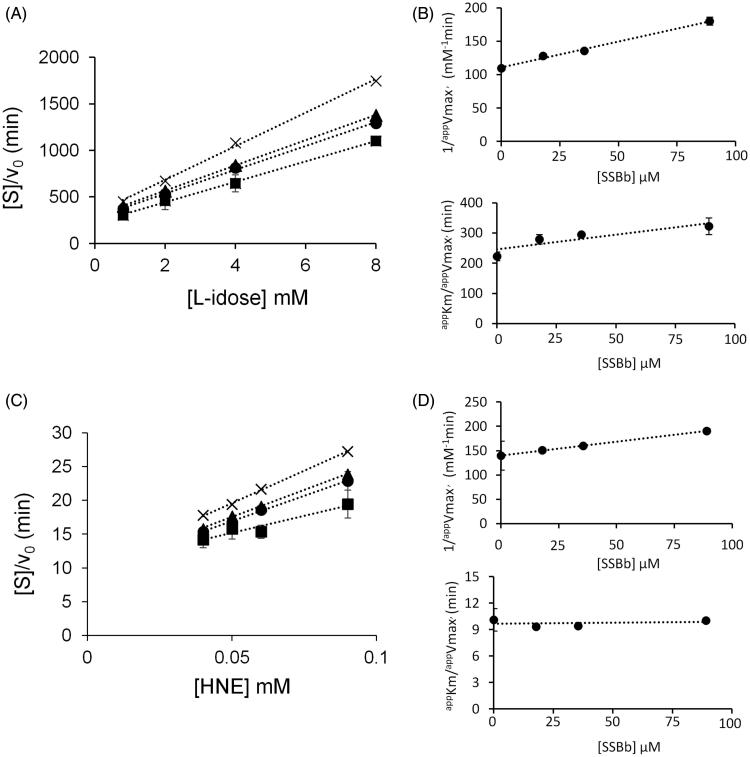
Kinetic characterisation of SSBb as *h*AKR1B1 inhibitor. *Panel* A and *Panel* C are the Hanes–Woolf plots obtained when the activity of the purified enzyme (8 mU) was measured at the indicated concentrations of the substrate, in the absence (■) or in the presence of the following inhibitor concentrations: (●) 17.8 µM, (▴) 35.5 µM, (×) 89 µM. *Panel* B and *Panel* D refer to the secondary plots of the slopes (1/*^app^V_max_*) and the ordinate intercept (*^app^KM*/*^app^V_max_*) of the relative Hanes–Woolf plot, as a function of the inhibitor concentration. *Panel* A and *Panel* B refer to l-idose; *Panel* C and *Panel* D refer to HNE. Error bars (when not visible are within the symbols size) represent the standard deviations of the mean from at least three independent measurements.

Figures 7 and 8 report a graphical analysis performed by the Hanes-Woolf plot of the kinetic data for the two inhibitors SSBa and SSBb, respectively. Table 2 reports the ternary enzyme-substrate-inhibitor complex dissociation constant (*K’_i_*) and the binary enzyme-inhibitor complex dissociation constant (*K_i_*) values. SSBa, acting as a mixed inhibitor, is characterised by rather low values of *K_i_* and *K’_i_* which are essentially identical for both substrates, thus justifying its higher efficiency as an ARI with respect to SSBb and, at the same time, its failure to exert any differential inhibitory action (Figure 6, *Panel* A). Conversely, SSBb acts on the two substrates by exhibiting a different inhibition model, behaving essentially as a purely non-competitive inhibitor with respect to l-idose, and displaying an uncompetitive mode of action with respect to HNE.

**Table 2.** Inhibition constants (µM) for SSBa and SSBb inhibiting either l-idose or HNE reduction. The value constants for SSBa and SSBb come from the steady state kinetic analysis described in Figures 7 and 8, respectively.

	SSBa		SSBb	
Substrate	*K’_i_* (µM)	*K_i_* (µM)	*K’_i_* (µM)	*K_i_* (µM)
l-idose	24 ± 2	66 ± 17	191 ± 11	176 ± 35
HNE	23 ± 4	65 ± 12	248 ± 19	n.a.

Data are reported as the mean ± SEM; n.a.: not applicable.

These results, which exemplify the predictable masking effect of an ARI over an ARDI, clearly indicate that either the isolation of single components or the availability of authentic standards are necessary to ascertain the ARDI features of molecule mixtures. In addition, these results enable SSBb to be included as a potential contributor to the differential inhibitory action of fraction 13 b. Unfortunately, the commercial SSBd standard (compound **4**) that we used to verify the possible contribution of this molecule to the differential inhibitory action of F13b, was revealed to be, in our hand, unsuitable. In fact, unlike other commercially available standard saponins used in the experimentation (i.e. SSBa and SSBb), the SSBd MS analysis failed to reveal either the expected compound or signs of possible saponin degradation products.

A final consideration emerging from the kinetic characterisation of SSBa and SSBb, is that although the most favourable and obvious feature for a molecule to behave as an ARDI towards two different substrates undergoing transformation would be the ability to express the inhibitory action only on one of them, different models of action of the inhibitor towards the two different substrates may also generate conditions for an inhibitory differential action. In fact, depending on the concentration values of the substrates in the assay, the enzyme may be more or less susceptible to inhibition depending on the inhibitory mechanism of action. A computer assisted simulation of the enzyme inhibition determined by different inhibitory mechanisms was thus performed at different substrate concentrations. The general equation (Equation (1)) was exploited, which was derived in ES steady state conditions for a general mixed type of non-competitive inhibition^26^, in which, besides the above defined $K_{i}$ and ${K'}_{i}\;$classical enzyme kinetic symbols are adopted:

(1) $$v_{0}=k_{cat}\left[E_{T}\right]/\left(1+\frac{K_{M}}{\left[S\right]}+\frac{K_{M}\left[I\right]}{K_{i}\left[S\right]}+\frac{\left[I\right]}{{K'}_{i}}\right)$$

**Figure 9.**
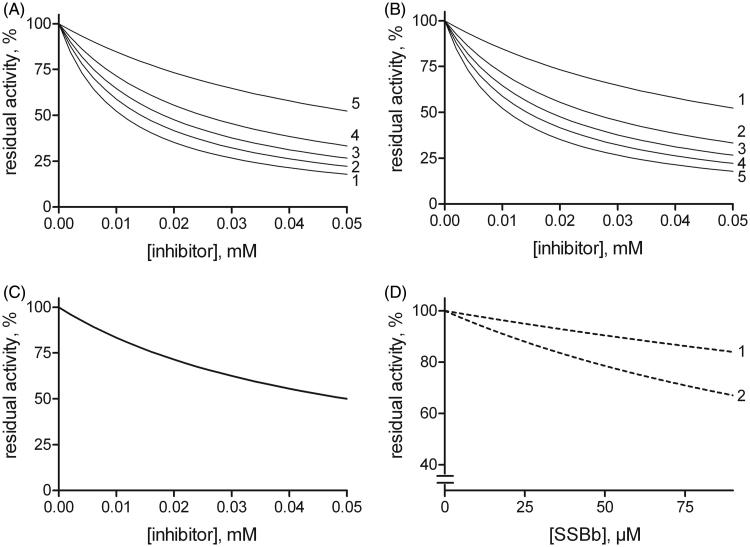
Effect of the substrate concentration on the susceptibility to inhibition for different inhibition models. Reaction rates (residual activity %) versus the inhibitor concentration at different fixed substrate levels were generated by computer-assisted simulation using a kinetic equation (see text, Equation (1) describing a general mixed inhibition model. *Panels* A, B and C: once a *KM* value of 1 mM had been arbitrarily fixed, the plots were generated for mixed type inhibitors exhibiting, *K_i_* (0.01 mM) ten-fold lower than *K’_i_* (0.1 mM) (*Panel* A), *K_i_* (0.1 mM) ten-fold higher than *K’_i_* (0.01 mM) (*Panel* B) or *K_i_* (0.05 mM) equal to *K’_i_* (*Panel* C). Curves 1–5 refer to fixed mM substrate concentrations of 0.01, 0.05, 1, 2, and 10, respectively. In *Panel* C the curves overlap. *Panel* D: A computer-assisted plot was generated to simulate the effect of SSBb making use of the inhibition constants on the activity of *h*AKR1B1 reported in Table 2 for HNE reduction (curve 1) and l-idose reduction (curve 2). The concentration of each substrate was fixed at a value which is the same one as adopted in Figure 6.

This enabled the residual activity versus the inhibitor concentration curves to be generated at substrate concentrations ranging from 1/10 *KM* to 10 *KM*. The inhibition mechanism types, defined on the basis of the relative values of the dissociation constants, were: competitive (*K’i/Ki* = 10^2^), uncompetitive (*K’_i_/K_i_*=10^−2^), and purely non-competitive (*K’_i_/K_i_*=1) (Figure 9, *Panels* A–C).

In the present case (Table 2), the *K’_i_* value for HNE reduction for SSBb is comparable to the constant referred to l-idose. However, the different mode of action towards the two different substrates shown by this inhibitor may generate a differential inhibitory action (Figure 9, *Panel* D). This result explains the observed differential inhibitory action in conditions, such as those adopted in Figure 6, that mimic the hyperglycaemia and oxidative stress, with the sugar levels in the mM range and HNE levels in the µM range.

## Conclusions

Following previous reports for Zolfino bean extracts that show its potential to inhibit AKR1B1^27^ and the recent observation for Zolfino bean that it exhibits an AKR1B1 differential inhibitory action^16^, the results emerging from the present work reveal the ability of some saponins to differentially inhibit the human aldose reductase enzyme. This was the case of SSBb and, possibly, of SSBd and/or an as yet unidentified saponin (compound **3**) present in the most differentially active sample derived from the Zolfino bean extract fractionation (Figure 2, *Panel* B, F13b).

The comparison between SSBb, acting as ARDI, and SSBa, acting as ARI is interesting. In this case, while the aglycone scaffold, which is common to both saponins, may represent the basic structural element of the interaction between the inhibitor and the enzyme, the sugar moiety of these molecules may modulate the differential inhibitory action.

Another aspect emerging from the present study is the possibility to extend the concept of differential inhibition to situations in which the inhibitor is active on different substrates, but through a different inhibitory model. In such a case, combined values of the concentration of the different substrates may generate differential inhibition.

It is clear that it is difficult to identify ARDIs in mixtures in which conventional ARIs are also present. In fact, the differential activity of an ARDI can easily be masked by the inhibitory action of the co-occurring ARIs, as is the case with SSBb and SSBa (Figure 6, F13a). This implies that for the search for new ARDIs to be successful, even modest indications of differential activity need to be considered while looking at any possible improvement in the mixture components separation.
